# Flood and Contain: An Optimized Repeal-Based Flooding Algorithm for Wireless Ad Hoc and Sensor Networks

**DOI:** 10.3390/s20205914

**Published:** 2020-10-20

**Authors:** Javier Gomez, Martha Montes-de-Oca, Jose Jaime Camacho-Escoto

**Affiliations:** 1 Telecommunications Deptartment, Engineering Faculty, National Autonomous University of Mexico, Mexico City 04510, Mexico; javiergo@comunidad.unam.mx; 2 Engineering Faculty, Panamerican University, Mexico City 03920, Mexico; mmontesd@up.edu.mx

**Keywords:** routing, wireless sensor networks, Ad Hoc, flooding, loT, resource discovery

## Abstract

Flooding is a simple yet reliable way of discovering resources in wireless ad hoc networks such as mobile ad hoc networks (MANETs), ad hoc sensors, and recently, IoT networks. However, its operation is resource-intensive, especially in densely populated networks. Several approaches can be found in the literature to reduce the impact of flooding. Many of these approaches follow a repeal-based operation, chasing and stopping further propagation of flooding packets once the target is found. However, repeal-based protocols might end up transmitting even more packets than the original flooding. This work characterizes a maximum repeal-flooding boundary beyond which it is counterproductive to chase the original flooding. We present the Flood and Contain (F&C) algorithm, a method that can quickly establish the maximum repeal-flooding boundary for each node while making no assumptions on the underlying network. F&C’s packet overhead increases linearly with the hop count up to the maximum repeal-flooding boundary, in which case there is no attempt to chase the original flooding. In this latter case, F&C generates only as many packets as the original flooding. Simulations show that, on average, F&C reduces the total flooding overhead (compared to traditional flooding) up to 35 percent once considering all possible destinations, with only a slight increase in resource discovery latency, and it outperforms all other repeal-based protocols, particularly for longer routes.

## 1. Introduction

Multihop Sensor Networks are a collection of sensors equipped with a wireless interface, each of which uses other nodes as relays. This type of network presents various advantages over traditional wired networks, mainly the ease of deployment. In sensor networks, it becomes necessary to measure variables over a vast region. Multihop Sensor Networks can also be found in the IoT networks by seeking to interconnect all things surrounding people with their environment to gather data from those things [1,2]. However, the one aspect that makes these networks both attractive and challenging is the nodes’ ability to communicate over long distances using other nodes as relays. In this sense, it is common that nodes seek a specific resource (such as a sensor with specific capabilities, a sink to transmit it or even finding a route to a specific node) before exchanging data [3].

Resource discovery can be achieved by using a traditional flooding technique where the resource is searched over all the possible nodes in the network. Although more complex route discovery protocols exist, traditional flooding remains the most widely used protocol in practice. In this protocol, a source node broadcasts a search packet looking for a specific target node. This packet is received only by nodes located within the source node’s transmission range. Upon receiving a search packet, a node adds its node ID to the packet header and retransmits it as soon as possible. This process continues until every node in the network has retransmitted the search packet. Once a search packet reaches the target node, it can respond to the source node, using unicast packets, through the reverse route stored in the packet header. Traditional flooding represents a robust and straightforward solution for route discovery, but it generates many packets, consuming much bandwidth [4]. Even though nodes in traditional flooding retransmit search packets once, many collisions may occur in densely populated networks due to a large number of nodes transmitting search packets simultaneously [5,6]. These collisions generate unnecessary retransmissions, increasing contention, and channel access delays for both control and data packets. This problem is known as the broadcast storm problem [4,7].

Various authors have proposed alternative methods to route discovery in ad hoc and sensor networks to address traditional flooding drawbacks. Although these protocols were originally designed for routing, these can also be applied to a resource discovery flooding. The following section presents a discussion of this issue in detail. However, most approaches attempt to repeal the propagation of flooded packets with chase flooding once either the source or target nodes reach each other. In general, protocols where the chase initiates at the destination are more efficient at stopping earlier the propagation of flooded packets. Repeal-based schemes can also be classified as bounded or unbounded, depending on the area nodes are allowed to chase and stop the propagation of the original flooding. Unbounded protocols allow any target node at any distance from the source node to chase the original flooding. On the other hand, bounded protocols allow a target node to chase ongoing flooding only if located within a given hop count from the source node. If the target node is not found, the maximum hop count is increased until, at a given hop count distance, the target node is finally found.

**Figure 1:**
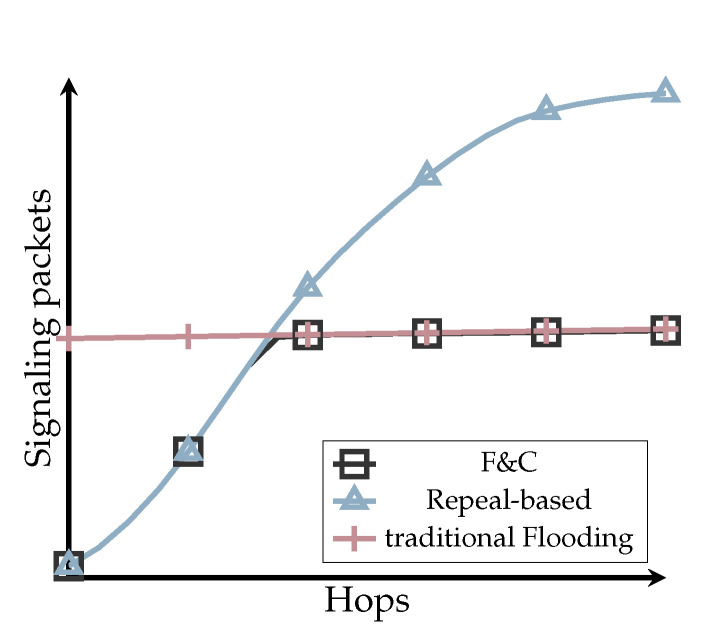
Behavior of Flood and Contain (F&C) vs. other schemes.

However, repeal-based protocols source or destination initiated, bounded or unbounded might end up transmitting even more signaling packets than the original flooding protocol they try to outperform. Figure 1 illustrates, in general terms, the behavior of most repeal-based flooding protocols vs. Flood and Contain (F&C). This figure is not the result of any simulation experiment, but a trending behavior observed in other works and the simulations performed at the end of this work (refer to Figure 13). In this figure, the number of signaling packets considering both the original flooding and chase packets is plotted against the hop-count separating source and destination nodes. As a reference, the number of packets generated by traditional flooding is shown as a constant number, indicating all nodes in the network always participated in the flooding process. As Figure 1 shows, repeal-based protocols are efficient only when the source and destination nodes are somewhat near to each other; otherwise, they can generate even more signaling packets than the original flooding. The main idea behind F&C is to have a mechanism that generates an overhead similar to repeal-based flooding schemes for near destinations, and behaves like traditional flooding for distant destinations. The critical point to achieve such a behavior is to allow each node to establish its optimized repeal-flooding boundary beyond which there is no attempt to chase and stop propagating the original flooding. Establishing and finding such an optimized repeal-flooding boundary is the key contribution of F&C and what sets this protocol apart from other repeal-based schemes.

The main features of the F&C algorithm are as follows:

1. F&C reduces the number of control messages over the whole network, thus saving energy and making the network more resistant to some Vampire Energy Depletion Attacks [8].
2. F&C protocol establishes a repeal-flooding boundary that delimits if the flooding is worth attempting to stop, allowing each node to decide whether it should initiate a chase flooding independently.
3. Experiments demonstrate that F&C achieves an optimized performance with as few as just one previous chase flooding, and its performance is relatively immune to variations in the network shape, node density, and mobility patterns.
4. F&C’s signaling overhead is proportional to the distance separating source and target nodes before the repeal-flooding boundary. This behavior makes F&C appeal to applications where the target node is located close to the source node, as in some IoT applications [1,2].
5. Considering all possible locations of the source and target nodes within the network, F&C generates up to 35 percent fewer signaling packets on average, compared with traditional flooding. Other repeal-based schemes might transmit even more signaling packets on average than traditional flooding.
6. F&C maintains a lower than or equal delay, compared with other repeal-based schemes. That is, it behaves at least as good as them while reducing the delay when the target node is far from the source node in terms of hops.

The rest of this paper is organized as follows. Section 2 presents relevant research related to reducing the overhead of flooding, particularly protocols based on chasing the original flooding. Section 3 presents a detailed description of the F&C algorithm. Section 4 describes how F&C allows each node to estimate its optimized repeal-flooding boundary. Section 5 describes the experiments conducted on the F&C algorithm as well as on other repeal-based schemes, and Section 6 presents the conclusions.

## 2. Related Work

Table 1 summarises the previous works made for trying to contain the overhead that traditional flooding generates. All protocols listed share a standard behavior; they chase the original flooding once the target node is found, attempting to stop further propagation of flooded packets in the rest of the network. Repeal-based protocols can be divided according to the entity that initiates the chase flooding into Broadcast-Repealing-Initiated in Source (BRIS) or Broadcast-Repealing-Initiated in the Destination (BRID). These protocols can also be divided into bounded, if the chase packet is bounded to an area (usually defined by a TTL), and unbounded if the chase packet can go along the whole network. Also, the protocols use different broadcasting speeds to allow the chase of the flooding messages. Such speed usually depends on the time required to transmit a packet δ, and the number of hops between the source and the retransmitting node *d*. Another distinctive feature of these protocols is the propagation strategy used during the initial and chase floodings. For example, protocols can use different propagation speeds for the initial and chase floodings, delay packets during the initial flooding so they can be reached by chasing packets, or might use one speed inside a ring and a different one outside the ring.

In L-B [9], the authors introduced the first attempt to contain the propagation of flooded packets by chasing the Resource Request (RQ) packets after the source node received a Resource Reply (RP) packet. The L-B protocol relies on different speeds for transmitting the RQ and the chase packet, with the chase packet speed being faster. In LHBA [11], they improved the chasing time by starting the chase in the destination rather than in the source. BERS [12] proposed a ring-search mechanism to reduce the energy consumption of TTL-Expanding Ring Search widely used by some reactive routing protocols. This mechanism was improved by BERS+ [13], where the ring expands without the need of retransmitting the original RQ from the source, but it resumes the expansion from the last ring. An update was performed in BERS* [15] by halving the waiting time in each ring, thus speeding up the route discovery. In [17], the chase stage occurs in the receiver rather than in the source node as performed earlier. In [14], the authors used the ring as a boundary to retransmit the RQ at two different speeds. Inside the ring, the RQ travels at speed $s_{1}$ while out of it, it travels at speed $s_{2}$ where $s_{1}\;>\;s_{2}$. AODV-PC [16] improved [14] by starting the chasing in the receiver. ABC [20] retransmits the RQ only to areas where it is more probable to find the destination node to prevent arbitrary flooding. This scheme has the disadvantage that it needs some previous information about routes, as well as topological information. A clustered version that combines BERS+ and MPR is presented in [21], where the authors reduced the number of broadcasts by selecting appropriate cluster heads.

Unbounded protocols (L-R, BERS+, TLRDA-C, AODV-PCABC, CMBERS+) do not establish a limit or boundary for the chase packets to contain the original flooding. As a result, these protocols can easily transmit more packets than traditional flooding alone. This behavior occurs when the distance between the source and destination is equal to or greater than the network radius, which is a frequent event. Bounded protocols (ERS, LHBA, BERS, BERS*, tBERS, BCIR, I-BERS) try to avoid the previous drawback by placing a ring-boundary defined by the source-destination hop count beyond which no chase packets can cross.

However, the performance of bounded and unbounded protocols might not always be what their authors expected. As Section 5 shows, bounded and unbounded protocols perform well only when the source and destination nodes are close to each other; otherwise, the added signaling of the original and chase flooding might easily become higher than the signaling of traditional flooding alone. As Section 4 shows, there exists an optimized repeal-flooding boundary, for any source-destination pair beyond it is counterproductive to chase the original flooding. Finding such boundary for chase packets is not a trivial task. Placing the boundary too close to the source makes chasing the original flooding unlikely as most destinations are usually located outside such boundary. Remember that only destinations found inside the repeal-flooding boundary contain the original flooding by using chase packets. On the other hand, placing the boundary farther away from the source increases the number of destinations allowed to chase the original flooding; however, adding up the overhead of the original flooding and chase packets may end up being higher than the overhead of traditional flooding.

This paper introduces Flood and Contain (F&C), a repeal-based flooding algorithm that can quickly establish the optimized repeal-flooding boundary for each node without making any assumptions on the underlying network. F&C falls into the BRID category that uses two separate flooding processes as most repeal-based schemes; however, opposite to other proposals, F&C does not attempt to chase the original flooding beyond the optimized repeal-flooding boundary. The evaluation section presents the conducted overhead and delays comparisons among the most significant repeal-based protocols listed in Table 1 and F&C to emphasize the improved qualities of the proposed algorithm.

There are other strategies proposed in the literature to reduce the signaling overhead of route discovery that do not consider the use of chase packets at any phase of their operation [22,23] and therefore are not directly comparable to F&C. For example, in [24,25], the authors proposed a probabilistic scheme for broadcasting search packets. Within such a scheme, upon receiving a search packet, a node rebroadcasts it with a specific probability (*p*). Probabilistic flooding reduces transmission redundancy, but it does not guarantee that all nodes receive the search packet, due to the fact that *p* is set to a fixed value [26]. In [27], the authors proposed a dynamic probabilistic scheme, in which each node uses a parameter for counting the number of times a packet was received, using it as a node density estimator. Other methods use neighbor-knowledge to form clusters and minimize the broadcast messages produced by flooding. One example is MPR (MultiPoint Relay) [28], in which each node periodically broadcasts hello messages to obtain a list of all its two-hop neighbors. Every node selects some symmetric nodes in its neighborhood called MultiPoint Relays, and only those nodes are selected to retransmit route search packets. In MPR, the number of retransmissions depends on the number of multipoint relays selected. MPR, used by OLSR (Optimized Link State Routing) [29], reduces duplicated retransmissions locally. However, maintaining this physical organization of the multipoint relays involves the use of a significant amount of signaling. The area-based method [22] is a different strategy for flooding packets in search of a destination, in which nodes use location information to decide whether or not to rebroadcast search packets. This method’s main disadvantage is that it relies on location hardware.

## 3. Flood and Contain (F&C)

The main goal of F&C is to stop further propagation of a flooding search once the target node is found, guaranteeing that the generated overhead is always less than or equal to the one generated by the original flooding. To achieve this, F&C uses two flooding events with different propagation speeds. During the initial flooding, a source node seeking a route to a node having a particular resource or feature floods the network with RQ packets. Upon receiving a RQ packet, a target node initiates a fast chase flooding to eat up the initial slow flooding packets to avoid unnecessary resource consumption in the rest of the network. For this operation to occur, it is necessary for the initial flooding to propagate more slowly than the chase flooding. To slow down the propagation of search packets during the initial flooding, nodes receiving an RQ packet do not retransmit it immediately, but keep it in their buffer for a short predetermined period ($\Delta_{t}$) before forwarding it. What sets F&C apart from any other repeal-based protocol is that nodes only launch a chase flooding if the reported hop-count to the source node is smaller than the node’s estimated repeal-flooding boundary. This condition guarantees that the aggregate overheard, considering the initial and chase flooding, never surpasses traditional flooding overhead. In case the reported hop-count to the source node is within the repeal-flooding boundary, the target node launches the chase flooding of RP packets. In this case, nodes attempt to retransmit RP packets as soon as they receive them. In case a node receives an RP packet before it has forwarded the previously received RQ packet, further propagation of both RQ and RP packets stops. This operation contains the area flooded with control packets during the resource discovery phase to a portion of the network only, as opposed to traditional flooding in which typically the entire network is flooded with signaling packets. Algorithm 1 defines the operation of the F&C protocol.
**Algorithm 1** F&C (S, N, D)
/*S: source node*//*D: Resource node set*//*N: node*/1:*S* initiates a slow flooding of RQ packets2:$N_{i}$ receives an RQ3:**if**$N_{i}\notin D$**then**4: $N_{i}$ reads delay flooding parameter $\Delta_{t}$5: $N_{i}$ waits $\Delta_{t}$ seconds6: $N_{i}$ forwards the RQ packet7:**else if**$N_{i}\in D$**then**8: **if** $N_{i}$ is reached after $N_{i}$’s repeal-flooding boundary **then**9:  $N_{i}$ replies to S (unicast) using the reverse route10: **else if** $N_{i}$ is located before $N_{i}$’s repeal-flooding boundary **then**11:  $N_{i}$ initiates a chase flooding of RP packets12: **end if**
13:**end if**14:$N_{i}$ receives an RP packet15:**if**$N_{i}$ has previously received an RP **then**16: $N_{i}$ does not forward the RP packet17:**end if**18:**if**$N_{i}$ has already received and forwarded the RQ packet **then**19: $N_{i}$ forwards the RP packet20:**else if**$N_{i}$ has the RQ packet in its buffer **then**21: $N_{i}$ does not forward the RQ and RP packets22:**end if**

**Figure 2:**
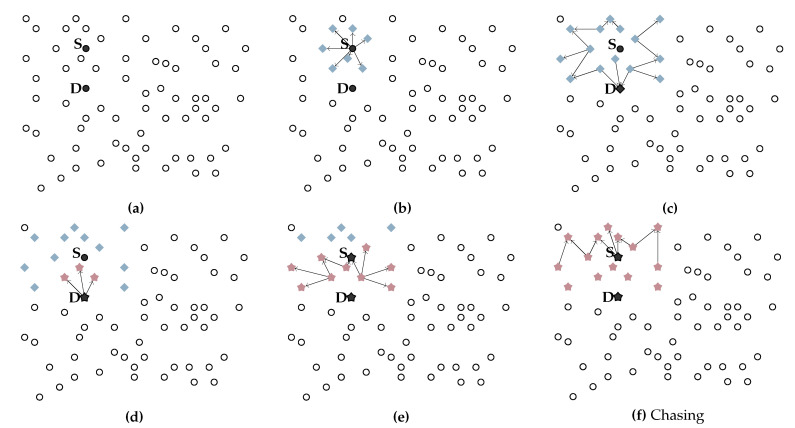
F&C operation. (**a**–**c**) Flooding stage; (**d**–**f**) Chasing stage.

Figure 2 graphically illustrates the behavior of the F&C protocol. Figure 2a shows source node *S* initiating flooding of RQ packets in search of a target node in *D*; blue diamonds illustrate nodes propagating RQ packets. Every time a node receives an RQ packet, it keeps the packet in its buffer for a predefined time interval ($\Delta_{t}$) before forwarding the packet again. Figure 2b,c show further propagation of RQ packets over time. Upon receiving the first RQ packet (see Figure 2c), a target node replies immediately with an RP packet (see Figure 2d) (this example assumed that the source node is within the target node’s maximum repeal-flooding boundary). The RP originates a chase flooding that propagates faster since nodes receiving an RP packet attempt to retransmit it as soon as possible. Figure 2d–f illustrate further propagation of RP packets, represented by red stars. Because the chase flooding moves faster, its propagation reaches and contains further propagation of the initial flooding process before it floods the entire network. Figure 2f shows how the chase flooding encompasses all the nodes covered so far by the original flooding. This operation allows F&C to reduce the area covered with route signaling packets, reducing the signaling overhead of the search process, compared with traditional flooding and other repeal-based protocols.

To gain some initial understanding of the problems found while attempting to repeal the initial flooding, we conducted a basic simulation of a standard repeal-flooding protocol where target nodes always attempt to contain the initial flooding, regardless of the distance. A detailed experimental study of repeal-flooding protocols versus F&C is presented later in Section 5. We implemented the repeal-flooding protocol in the ns-3 network simulator [30] and conducted a series of experiments to observe its behavior and present some areas of opportunity to improve its performance. Simulations used the standard IEEE 802.11g and randomly placed 300 nodes in a 3500 × 3500 m${}^{2}$ network. The transmission range is 250 m. The simulations consisted of allowing one node to find a route to each of the remaining 299 nodes. Similar to traditional flooding, there was no previous knowledge about the network’s state for any of the 299 simulations. For each simulation, the number of signaling packets needed to discover the target node was added, including RQ (i.e., original flooding) and RP packets (i.e., chase packets). There was no chase boundary in these experiments, so the target node always initiated a chase flooding after receiving an RQ packet to illustrate the need for such a boundary.

**Figure 3:**
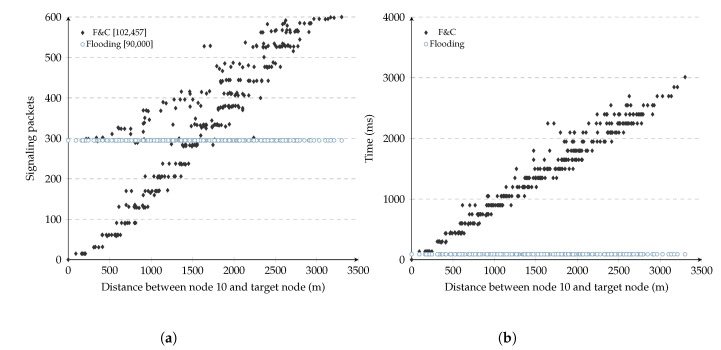
Signaling packets and route delay from node 10 to 299 nodes in the network. (**a**) Signaling packets; (**b**) Route delay. RQ: 150 ms, RP: 0 ms.

Figure 3a shows the number of signaling packets generated by this repeal-flooding protocol versus the distance separating source and target nodes for each of the 299 experiments (each point in the figure represents one such experiment). In these experiments, each node used a fixed 150 ms delay before attempting to forward RQ packets during the initial flooding. As a reference, the figure also shows the number of packets generated by traditional flooding, which remained constant at 299 packets per experiment. The number inside the square brackets in this figure shows the aggregate signaling overhead for the 299 experiments for either the repeal-flooding protocol and traditional flooding. On the other hand, Figure 3b shows the delay it took the repeal-flooding protocol to reach each of the 299 nodes versus the distance between the source and target nodes. We refer to the three key observations resulting from these figures as *distance*, *escape*, and *delay* issues, and explain them below.

### 3.1. Distance

Referring to Figure 3a, it shows that the number of signaling packets generated by the repeal-flooding protocol increased linearly as the distance separating source and target nodes increased. This increment is the expected behavior since longer distances involve more hops and more signaling packets while attempting to find the target node (e.g., slow flooding) and contain its propagation with a chase flooding (e.g., fast flooding). This figure shows how particularly effective at containing the number of nodes involved in the route search process a repeal-flooding protocol is whenever source and target nodes are somewhere near each other. Unfortunately, the same figure shows that in some experiments, the repeal-flooding protocol can generate even more signaling packets compared with traditional flooding when longer distances are present. This problem is referred to as the distance problem and is addressed by the F&C protocol in Section 4. Finally, please note that the distance between the source node and the furthest node in Figure 3a depends on the source node’s position (node 10). In this figure, node ten was located in mid-position between the center and boundary of the network.

### 3.2. Escape

The escape issue is related to the points found above the 300 packet level in the 0–1200 m range in Figure 3a. At first, these points seemed odd since they were supposed to follow the trend of other points at that distance range. Looking deeper at the experiments leading to these points, we found that a collision of packets belonging to the chase flooding had caused an incorrect reception of an RP packet by one node, which caused it to transmit a queued RQ packet. This escaped RQ packet eventually flooded the entire network with packets belonging to the original flooding. This problem is not exclusive to repeal-flooding protocols; in fact, it appears in most route discovery protocols using the IEEE 802.11 MAC standard, even affecting traditional flooding as well since collisions of RQ packets may prevent the target node from being reached.

A simple solution to the collision problem is to change the added delay during the initial flooding to be a random variable with mean $\Delta_{t}$. Also, the chase flooding phase might add a random delay, which might be chosen to be as small as possible, in order to avoid delaying the chase of the original flooding. This random delay will reduce the probability that two nodes transmit RQ or RP packets simultaneously, thus avoiding a collision.

### 3.3. Delay

Figure 3b shows the delay it took the repeal-flooding protocol to reach each of the 299 nodes versus the distance between the source and target nodes. As the figure shows, the resource discovery delay increases monotonically as the distance between the source and target nodes increases. In most repeal-flooding protocols, each node delays retransmissions of RQ packets during the initial flooding by $\Delta_{t}$ seconds, but how long should that delay take? If the delay is too short, the chase flooding might not stop propagating the initial flooding before it covers the entire network. If the delay is too long, then the overall time it takes for the source node to reach the target node can get to the point that it becomes unbearable for the application. The value of $\Delta_{t}$ should last longer than the accumulated delay of the resource discovery by the time it reaches the target node, which depends on the number of hops in the route and the MAC layer technology used. Simulations used to evaluate F&C use the IEEE 802.11g standard, and in [31], they found forwarding delays in the range of 2–10 ms for one hop only (including channel contention and packet transmission). Simulations in Figure 3b used a value of $\Delta_{t}$ set to 150 m, so the chase flooding was able to contain the propagation of the initial flooding, even in routes having 10–15 intermediate hops. The value of $\Delta_{t}$ can also be easily adjusted depending on whether or not the chase flooding contained the propagation of the first flooding before it propagated in the entire network. A detailed analysis of $\Delta_{t}$ is presented in Section 5.1.

## 4. Repeal Flooding Boundary

As Figure 3a shows, whenever source and target nodes are located far away from each other, this causes most repeal-based protocols to generate more signaling packets than traditional flooding. There is even the possibility repeal-based schemes generate twice as many packets (the case in which both the initial and chase flooding covered the entire network, respectively). It would be beneficial if F&C sometimes did not initiate the chase flooding when the source and target nodes are far from each other, and let the target node reply to the source node through the reverse route using unicast packets, as in traditional flooding. The target node’s inaction causes the propagation of RQ packets to continue, flooding the entire network with RQ packets just as in traditional flooding. As such, there is a repeal-flooding boundary (FB), for every node beyond the use of chase flooding is disadvantageous (i.e., F&C generates more signaling packets than traditional flooding). For the experiment represented in Figure 3a, the repeal-flooding boundary for most nodes can visually be placed at about 1200 m away from source node 10, the distance at which F&C begins generating more signaling packets than traditional flooding.

### 4.1. Finding the Repeal-Flooding Boundary

**Figure 4:**
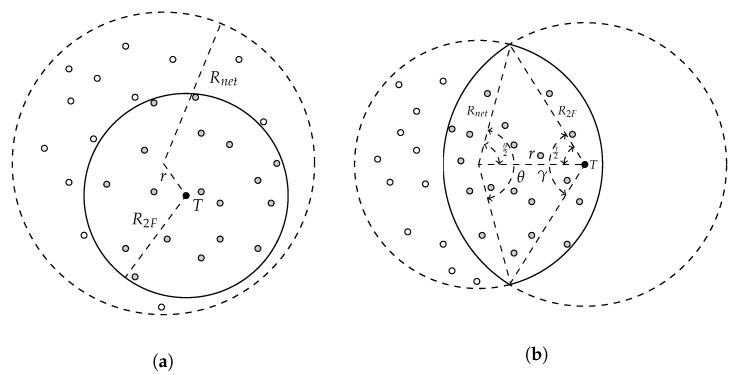
Maximum area (solid line) where launching a chase flooding generates similar overhead compared with traditional flooding. (**a**) Disk shaped network; (**b**) Lens shaped network.

Let us start by considering the case in which *N* nodes are distributed homogeneously in a disk-shaped network of radius $R_{net}$, as shown in Figure 4 (the evaluation section will show that these network assumptions can be relaxed with little impact on the overall protocol behavior). Now, we define $F_{1F}$ and $F_{2F}$ as the number of signaling packets generated by the initial (1F) and chase (2F) flooding phases of F&C. On the other hand, let $F_{TF}$ account for the number of signaling packets generated by traditional flooding, which is assumed to be N−1, the case in which all nodes are connected. F&C works better (i.e., generate fewer signaling packets compared with traditional flooding) as long as

(1) $$F_{1F}+F_{2F}<F_{TF}$$

$F_{1F}$ is the same as $F_{2F}$ since the number of signaling packets generated by the chase flooding propagates only through nodes that were already visited by packets belonging to the first flooding. Because nodes are assumed to be distributed homogeneously in the network with density ρ, the expected number of nodes in a given area *A* is ρA. Thus, finding $F_{1F}$ and $F_{2F}$ is equivalent to finding the number of nodes in the area covered by signaling packets during the original flooding and posterior chase flooding (e.g., $\rho A_{1F}$ and $\rho A_{2F}$).

Since the goal of F&C is not to surpass the number of signaling packets generated by traditional flooding, there is a limit to how distant source and target nodes can be from each other. To find this limit, we assumed a worst-case scenario, in which the number of signaling packets generated by F&C is the same as in traditional flooding $F_{TF}$, meaning that:

(2) $$F_{1F}+F_{2F}=F_{TF}$$

or equivalently

(3) $$A_{1F}+A_{2F}=A_{TF}$$

While traditional flooding always floods the entire network, the shape of the area flooded with RQ and RP packets changes depending on the source and target nodes position within the network in F&C. In general, two scenarios appear: (i) the flooded area takes the shape of a disk centered at the target node; and (ii) the flooded area takes the shape of a lens resulting from intersecting two disks. Section 4.1.1 explains Figure 4, which illustrates these two scenarios.

#### 4.1.1. Disk-Shaped Scenario

Figure 4a illustrates the case in which the shape of the area covered with signaling packets belonging to the first and chase flooding is a disk with the target node located at its center. The equation below derives from Equation (3):

(4) $$2\pi R_{2F}^{2}=\pi R_{net}^{2}$$

Then, the maximum radius of the chase flooding (i.e., repeal-flooding boundary), in which the combined signaling of the first and chase flooding generates the same number of signaling packets as traditional flooding, becomes:

(5) $$FB=\frac{1}{\sqrt[{}]{2}}R_{net}$$

The disk shape remains as long as the source node is within 0 and 0.3$R_{net}$ from the center of the network.

#### 4.1.2. Lens-Shaped Scenario

As the source node moves away from the network’s center (i.e., between $0.3R_{net}$ and $R_{net}$), the previously described disk shape increases its radius up to a point where it intersects the network boundary, creating the lens shape shown in Figure 4b. The lens shape results from the overlapping of two disks. The first disk is the network boundary, having an $R_{net}$ radius, while the chase disk’s center is at the location of the target node, and its radius is the distance between the source and target nodes (see Figure 4b). The lens area’s size depends on the distance separating the target node from the network’s center, denoted as *r*, as well as the angles between the origins and intersection points of the two disks, named α and γ (see Figure 4b). The lens region area results from adding two segments named $S_{1}$ and $S_{2}$, which can be computed as follows:

$$S_{1}=\frac{1}{2}R_{net}^{2}\left(\theta -\sin\theta \right)$$

$$S_{2}=\frac{1}{2}R_{2F}^{2}\left(\gamma -\sin\gamma \right)$$

with both θ and γ expressed in radians, therefore the overlapping area (i.e., $S_{1}+S_{2}$) becomes:

(6) $$A_{1F}=A_{2F}=\frac{1}{2}R_{net}^{2}\left(\theta -sin\theta \right)+\frac{1}{2}R_{1F}^{2}\left(\gamma -sin\gamma \right)$$

Applying the law of cosines to either triangle in Figure 4b, these angles can be computed as follows:

(7) $$\theta =2\sec(\frac{r^{2}+R_{net}^{2}-R_{2F}^{2}}{2aR_{net}})$$

and

(8) $$\gamma =2\sec(\frac{r^{2}+R_{2F}^{2}-R_{net}^{2}}{2aR_{net}})$$

Similar to the disk-shaped case, the maximum radius of the chase flooding (i.e., repeal-flooding boundary), in which the combined signaling of the first and chase flooding generates the same number of signaling packets as traditional flooding, becomes:

(9) $$A_{1F}+A_{2F}=A_{net}$$

or equivalently

(10) $$2\left(S_{1}+S_{2}\right)=A_{net}$$

Solving for FB provides:

(11) $$FB=\frac{1}{\sqrt[{}]{2}}R_{net}+0.64\left(r-0.3\right)$$

**Figure 5:**
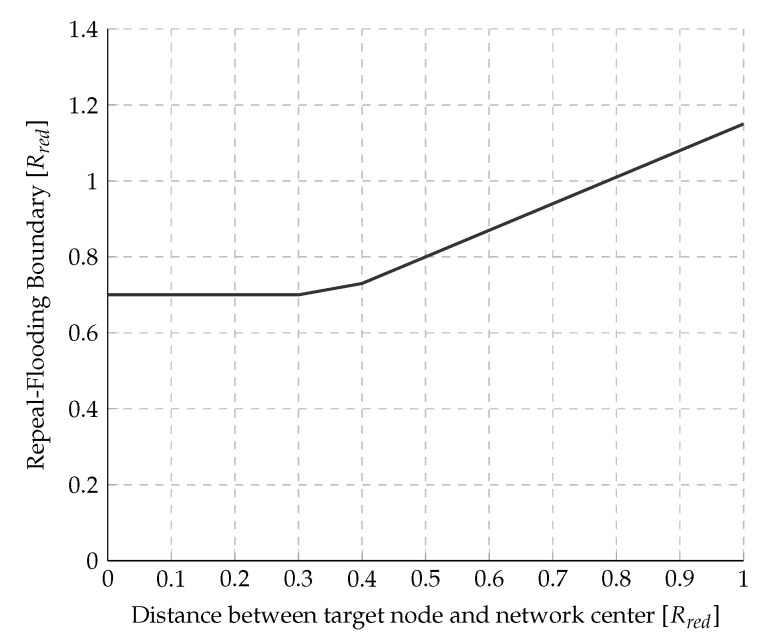
Repeal-flooding boundary versus target node’s distance to the network’s center.

Finally, the repeal-flooding boundary (FB) becomes:

(12) $$FB=\left\{\begin{array}{cc} \frac{1}{\sqrt[{}]{2}}R_{net}, & \;0<r<0.3R_{net}\\ \frac{1}{\sqrt[{}]{2}}R_{net}+0.64\left(r-0.3\right), & \;0.3R_{net}<r<R_{net} \end{array}\right.$$

where, again, *r* is the target node’s distance to the center of the network, and $R_{net}$ is the radius of the network. The next section will explain how any node can estimate both distances in practice, so each node can compute its flooding boundary using Equation (12). Figure 5 shows how the flooding boundary (i.e., the point where the signaling overhead of F&C equals the signaling overhead of traditional flooding) changes as the source node moves away from the network’s center. As this Figure shows, this boundary remains constant at $\frac{1}{\sqrt[{}]{2}}R_{net}$ when the target node is within 0 and $0.3R_{net}$ from the network’s center, which corresponds to the case where the overlapping area is disk-shaped. Beyond $0.3R_{net}$, the overlapping area is a lens, and the value of the repeal-flooding boundary increases linearly with the distance up to a value of $1.17R_{net}$, where the target node reaches the edge of the network. So far, in the derivation of the optimized repeal-flooding boundary, distance was considered in the analysis rather than hop count since the latter depends on both the distance and node density, which is more challenging to deal with. However, hop count units will help to establish the optimized repeal-flooding boundary in practice in Section 4.1.3.

The graph shown in Figure 5 is used as follows: Whenever a source node needs a route, it launches slow flooding to search for the target. Upon being reached, the target node uses its estimated repeal-flooding boundary, according to Figure 5, to decide whether or not it should launch a chase flooding. In case the accumulated route hop-count included in the RQ packet falls below the target node’s repeal-flooding boundary, the target node launches a chase flooding to stop further propagation of the initial flooding. This chase flooding results in an overall reduction of the signaling overhead of F&C compared with traditional flooding and other repeal-based protocols, as shown later. On the other hand, if the route hop-count is beyond the target node’s repeal-flooding boundary, no chase flooding is launched, and the initial flooding continues until it propagates through all nodes in the network. In this case, the number of signaling packets generated by F&C is N−1, similar to traditional flooding.

#### 4.1.3. Finding the Repeal-Flooding Boundary in Practice

**Figure 6:**
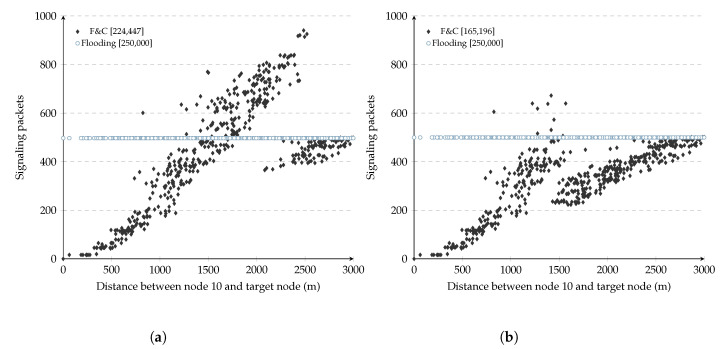
Signaling packets from node 10 to each target node, considering different repeal-flooding boundary (FB) values. (**a**) Flooding Boundary = 11; (**b**) Flooding Boudary = 7.

The previously described operation is challenging to carry out in practice. In a real wireless ad hoc and sensor network, node density and the network’s shape and size are generally unknown, let alone the target node’s position within the network [32,33]. Consequently, in practice, it is not straightforward for any node to estimate its repeal-flooding boundary accurately. Figure 6 shows the number of signaling packets generated by F&C versus the distance separating source and target nodes and two different arbitrary repeal-flooding boundary values (i.e., 11 and 7 hops). The simulations settings considered the IEEE 802.11g and randomly placed 500 nodes in a 3500 × 3500 m${}^{2}$ network. The transmission range is 250 m. In Figure 6a, all nodes located within 11 hops from the source node initiated chase flooding. In many cases, this operation resulted in more signaling packets than traditional flooding (i.e., points found above the 500 packet line). On the other hand, Figure 6b shows the results when nodes initiated chase flooding only if reached within seven hops from the source node. In this case, the vast majority of points are below the 500 packet line. These figures illustrate how the repeal-flooding boundary value estimated by each node impacts the performance of F&C.

**Figure 7:**
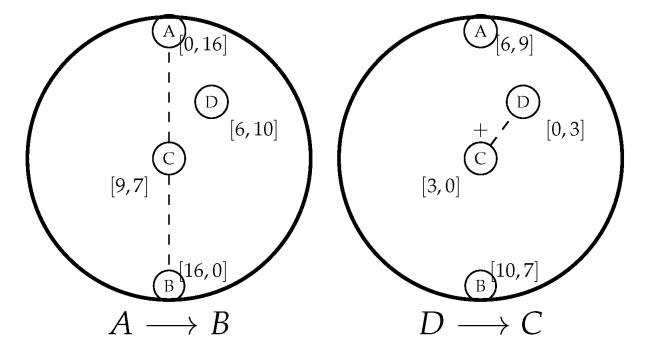
Estimating the repeal-flooding boundary.

Nodes need some information regarding the network’s size and their location within it to estimate their repeal-flooding boundary. Initially, we assumed that nodes have an infinite repeal-flooding boundary, meaning they will chase any incoming flooding while estimating a more realistic boundary. Now, for node *i* (represented by $n_{i}$) to be able to estimate its repeal-flooding boundary $FB_{i}$, $n_{i}$ needs a clue that helps it compute $FB_{i}$ with accuracy while requiring minimal intervention from the other nodes. In F&C, this clue takes the form of an unbounded version of F&C protocol; this is, a target node that has not previously estimated its repeal-flooding boundary launches a chase flooding after receiving an RQ packet. This initial behavior of F&C is similar to an unbounded repeal-based protocol. Once a target node chases the initial flooding with RP packets, other nodes overhearing both RQ and RP packets can estimate their repeal-flooding boundary according to the procedure described below. By doing this, they can make the best decision as to whether or not to chase the original flooding in the future. The evaluation section shows that it takes as few as one chase flooding in the best of cases, and only a few chase floodings on average, for all nodes in the network to estimate their repeal-flooding boundary with reasonable accuracy. Figure 7 illustrates the way nodes estimate their repeal-flooding boundary. This figure illustrates two different resource searches: a long distance route resource (16 hops) from node *A* to node *B* (Figure on the left), and a short distance route resource (3 hops) from node *D* to node *C* (Figure on the right). In both resource searches, we assumed that each node in the network increases by one #RQ or #RP counter every time they forward those packets, respectively. The square brackets next to each node in Figure 7 show the pair [#RQ,#RP] as a result of using F&C for each resource search.

Now $n_{i}$ can estimate the radius of the network ($R_{i}$) and its distance to the center of the network ($r_{i}$) as follows:

(13) $$R_{i}\approx \frac{\#RQ+\#RP}{2}$$

(14) $$r_{i}\approx R_{i}-min\left[\#RQ,\#RP\right]$$

In simple terms, each node guesses if it lies somewhere along the shortest route resource connecting the source and target nodes. The diameter of the network is thus #RQ + #RP. Then, it follows that the network radius is just half the diameter, and the node’s distance to the center of the network is the estimated network radius minus the minimum of either #RQ or #RP. A node can now use Equation (12) to establish its repeal-flooding boundary. Notice that while the units on both axes in Figure 5 are given in terms of fractions of $R_{net}$, the ratio $\frac{r_{i}}{R_{net}}$ used in the horizontal axis can be easily approximated as $\frac{r_{i}}{R_{net}}$ in hops. For each new search, $n_{i}$ will update the value of $R_{i}$ and $r_{i}$ in hops using the largest estimated value so far, respectively, which will modify the latest value of the repeal-flooding boundary.

Table 2 shows the computation of $R_{i}$ and $r_{i}$ after each resource search for the left case in Figure 7 (where node D seeks node C).

In contrast, Table 3 shows the same parameters for the case when node A is seeking node B. As the tables show, the estimate of $R_{i}$ and $r_{i}$ is accurate for all nodes due to the long route, but not accurate as a result of the short route. Now, it is just a matter of letting each node use the largest estimated value of either $R_{i}$ and $r_{i}$ from the two tables in this example to improve its repeal-flooding boundary accuracy.

As Table 4 shows, it takes as few as one long resource search using F&C for all nodes to accurately estimate their repeal-flooding boundary.

**Figure 8:**
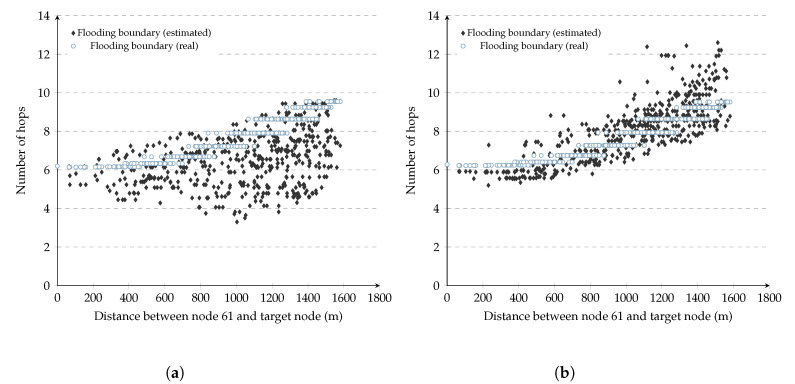
Repeal-flooding boundary (optimized vs. estimated). (**a**) After 2 resource searches; (**b**) after 5 resource searches.

Figure 8 shows how each of the 500 nodes computed its repeal-flooding boundary after two and five previous resource searches using F&C. The same figures show, as a reference, the optimized repeal-flooding boundary for each node (obtained manually). As can be observed in these figures, while repeal-flooding boundary inaccuracies appear after two resource searches, after five resource searches, the majority of nodes estimated a repeal-flooding boundary value that is within two hops of their optimized value. This behavior demonstrates the ability of F&C to set an accurate repeal-flooding boundary value at each node with minimal signaling cost.

## 5. Experiments and Results

We implemented the F&C protocol in the ns-3 network simulator [30]. Various metrics affecting the performance of F&C protocol were studied, including the propagation speed of the initial flooding ($\Delta_{t}$), repeal-flooding boundary estimation accuracy, and the operation of F&C under non-ideal conditions. Finally, at the end of this section, a comparison between relevant repeal-based protocols and F&C is also discussed. Table 5 shows the simulations setting used in the experiments.

### 5.1. Choosing the Speed of the Initial Flooding, $\Delta_{t}$

**Figure 9:**
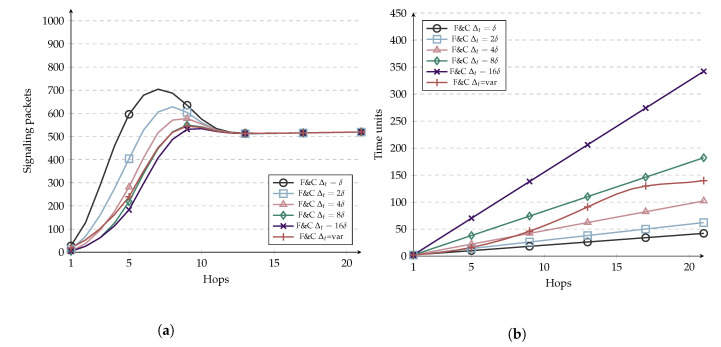
Comparison of F&C with different *d*. (**a**) Signaling overhead; (**b**) Resource discovery delay.

A parameter that can be tuned in F&C and impacts its performance is $\Delta_{t}$. We performed simulations to evaluate the impact of selecting such a parameter on signaling overhead and resource discovery time. Simulation parameters are shown in Table 5 and results are shown in Figure 9a,b. The simulations comprise the evaluation of five different fixed $\Delta_{t}$ values and a variable version of $\Delta_{t}$. The variable version behaves as follows: When the hop count between the source and target nodes is smaller than the repeal-flooding boundary, $\Delta_{t}$ is the hop count from source to the current node times δ where δ is defined as the time necessary to transmit one packet from two adjacent nodes. Else, $\Delta_{t}=1\times \delta$. As Figure 9a,b show, the use of a greater $\Delta_{t}$ results in longer delays but also decreases signaling overhead since the initial flooding is contained earlier. On the other hand, for smaller values of $\Delta_{t}$, the overall delay decreases since nodes wait less time to retransmit the RQ. However, the initial flooding is contained farther, thus increasing the signaling overhead. Variable $\Delta_{t}$ shows an exponential behavior in the delay before hop 16, behaving just like $\Delta_{t}=\delta$ afterward. Notice that variable $\Delta_{t}$ behaves similarly to $\Delta_{t}=8\delta$ in terms of signaling overhead, but variable $\Delta_{t}$ outperforms it in terms of delay considering the whole distance range.

### 5.2. Estimating the Repeal-Flooding Boundary

**Figure 10:**
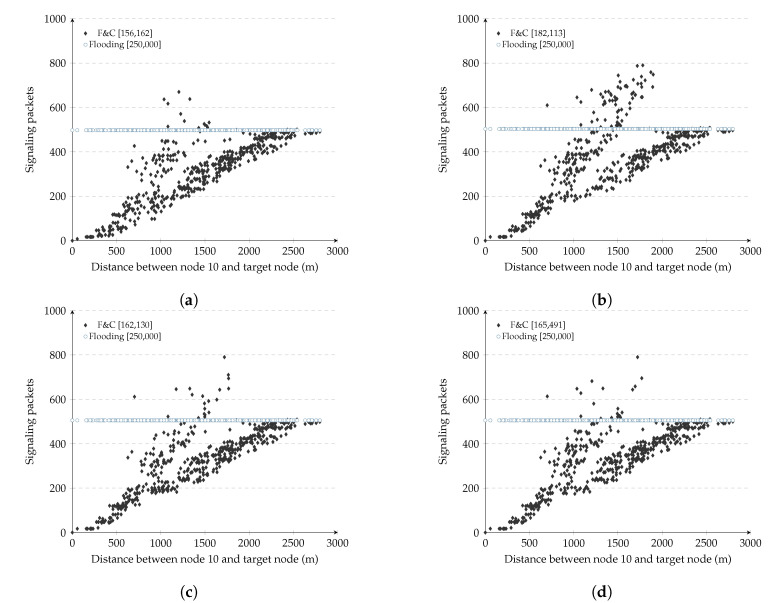
F&C: Signaling packets for different repeal-flooding boundary values. (**a**) Using optmized repeal-flooding boundary; (**b**) After 2 resource discovery searches; (**c**) After 5 resource discovery searches; (**d**) After 10 resource discovery searches.

Figure 10 shows the signaling overhead versus distance of F&C with the repeal-flooding boundary. The experiments consisted of one node searching for a route to each of the remaining 499 nodes, in this case. As a reference, Figure 10a shows the case in which each node manually learned about its optimized repeal-flooding boundary value in advance. This case represents the best performance F&C can achieve and a benchmark for comparison. In Figure 10b, nodes use only the first two resource searches, using the unbounded version of the F&C protocol, to estimate their repeal-flooding boundary, as discussed in Section 4.1.3 (the two routes were selected randomly). Once every node estimated its repeal-flooding boundary, its value remained constant for the remaining 497 resource searches. Figure 10b shows that it took F&C 182,113 signaling packets for one node to find a route to the other 499 nodes under these conditions. This result represents 27 percent fewer signaling packets compared with traditional flooding. Figure 10c,d show the signaling overhead versus the distance of F&C after nodes use the first 5 and 10 resource searches only to estimate their repeal-flooding boundary, respectively. Figure 10c shows how F&C achieved a 35 percent signaling reduction compared with traditional flooding, which represents only two percent higher than the performance when nodes use the optimized repeal-flooding boundary (see Figure 10a). This signaling reduction is the result of nodes estimating a more accurate repeal-flooding boundary value. Figure 10d, on the other hand, did not improve the results from Figure 10c, even if the nodes used the first ten resource searches to estimate their repeal-flooding boundary. Looking into the source and target nodes’ location for resource search 6 to 10 in Figure 10d, we found that they corresponded to shorter routes when only a few nodes updated their repeal-flooding boundary compared with the value they had after five resource searches. This result is a reminder that to estimate an accurate repeal-flooding boundary, the length of the route of a resource search is more important than the number of resource searches. Overall, Figure 9 clearly shows the ability of F&C to quickly achieve an optimized performance with a minimal number of previous resource searches.

### 5.3. Density, Mobility and Shape Concerns

**Figure 11:**
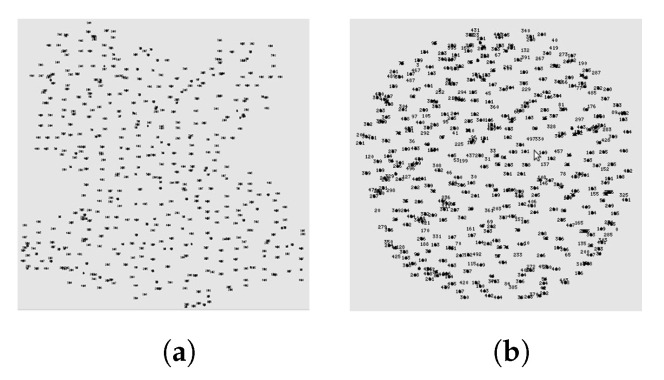
F&C: Disk and irregular network shape. (**a**) Irregular Shape; (**b**) Disk Shape.

So far, the presentation and evaluation of F&C had been considered a disk-shaped network with homogeneous density and no mobility. Consequently, various questions immediately arise: What would happen if the density were not homogeneous? What would happen if the shape of the network were irregular? Would the protocol still operate in mobile environments? Table 6 shows the answers to these questions. This table shows the percentage of signaling packets used by F&C operating under ideal and non-ideal conditions versus the number of packets generated by traditional flooding. As for the shape of the network, we used the irregular shape shown in Figure 11a. For the density experiment, we used a disk-shaped network divided into two halves: the right half had higher node density (i.e., 300 nodes), whereas the left half had lower node density (i.e., 200 nodes). This node arrangement was selected, so all nodes remained connected even in the network’s left half. Finally, for the mobility experiment, nodes moved at 1 m/s using the random-waypoint mobility model. In all ideal and non-ideal experiments, the network area and the number of nodes were kept constant, and nodes estimated their repeal-flooding boundary using the first five resource searches only. The experiments consisted again in computing the overall signaling overhead of one node seeking a route to the other 499 nodes, and the results are shown as the percentage of signaling packets generated by traditional flooding that needed 250,000 packets (i.e., 500 × 500).

**Figure 12:**
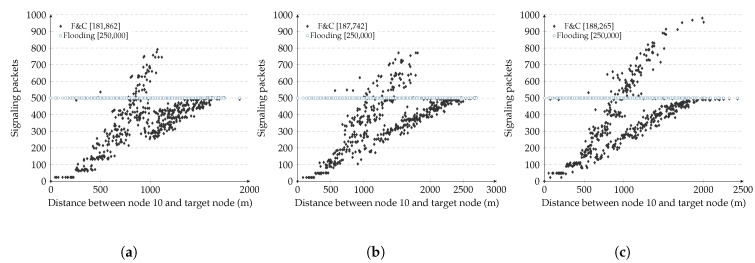
F&C under non-ideal conditions. (**a**) Irregular network shape; (**b**) Mobility: After nodes moved during 1 min; (**c**) Mobility: After nodes moved during 10 min.

The results in Figure 12 and Table 6 show that even if F&C always achieves its best performance when it operates under ideal network conditions. The performance obtained under non-ideal conditions is not very different from ideal conditions and was 25 to 40 percent better than traditional flooding in all non-ideal scenarios. The irregular-shaped experiment showed that nodes estimated a non-optimized diameter of the network due to the non-disk shape. In turn, this produced an erroneous estimate of the repeal-flooding boundary. However, the final result shows that the signaling overhead was 7 percent higher than the signaling overhead obtained under ideal conditions. As for the density experiment, F&C performance depended on node density around the source node. Let us keep in mind that F&C achieved its best performance when the target node was closer to the source, so when node density around the source node was higher than the average density, F&C achieved better results than when it operated under homogeneous density conditions. An opposite trade-off took place when fewer nodes surrounded the source node. As for mobility, results show that more important than how fast nodes moved is when was the last time that nodes updated their repeal-flooding boundary. As can be observed in the table and in the figure, as long as there is a fresh estimate of the repeal-flooding boundary (i.e., after 60 s), there is little performance degradation. Even after 10 min of the last repeal-flooding boundary estimate, F&C performance remained better than traditional flooding. In summary, even if there is performance degradation, the results in Table 6 and Figure 12 show F&C as a robust and reliable protocol under a diverse array of network conditions.

### 5.4. F&C versus Other Repeal-Based Flooding Protocols

**Figure 13:**
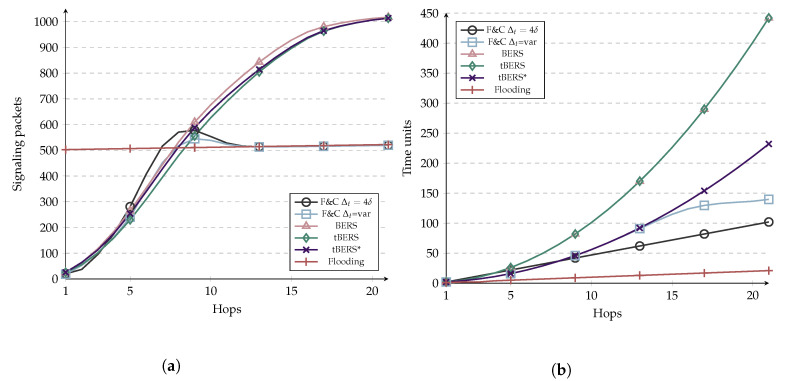
Comparison of flooding contention protocols. (**a**) Signaling overhead; (**b**) Route discovery delay.

Now we present a comparison between F&C and other repeal-based flooding schemes. BERS [12], tBERS, and tBERS* [17] were selected for this analysis since they are protocols comparable to F&C since they chase the initial flooding once the source and target nodes find each other. To evaluate the performance of such protocols and compared them to F&C, we performed simulations in SimPy [34]. Figure 13 shows the results of the simulation of such protocols in terms of signaling overhead and resource discovery delay, respectively. The simulation parameters are the same as before (see Table 5).

The plots in Figure 13a show that BERS generates a ring search that minimizes the flooded area for nodes close to the source. Nevertheless, the ring search technique increases the resource discovery delay shown in Figure 13b. tBERS, on the other hand, repeals the flooding in a target node, reducing the number of signaling packets needed to find it. Despite this, the delay remains the same as in BERS. An improvement was made in tBERS* by reducing 50% the retransmission delay, thus halving the resource discovery delay while slightly increasing the signaling overhead. The plots in Figure 13a show that the signaling packets generated by BERS, tBERS, and tBERS* can surpass the number generated by traditional flooding as the route hop count increases. In the worst case, these protocols can generate twice as many signaling packets than traditional flooding when the source and target nodes are far from each other.

F&C behaves similarly to the BERS family of protocols before the route hop-count to the resource reaches the target’s repeal-flooding boundary and behaves similar to traditional flooding afterward (i.e., no attempt is made to chase the initial flooding). As a result of this behavior, F&C’s signaling increases with the hop count, up to a point it remains similar to traditional flooding. Figure 13, more than any other figure, shows the key distinctive feature of F&C over other repeal-based protocols. In terms of delay, F&C with $\Delta_{t}=4\delta$ behaves linearly with the route hop count to the resource while being slower than tBERS* before nine hops, improving afterward. When F&C uses variable $\Delta_{t}$, i.e., $\Delta_{t}=var$, the delay behaves as tBERS* before 13 hops and starts improving after that point. Notice that F&C with $\Delta_{t}=var$ improves F&C with $\Delta_{t}=4\delta$ in terms of overhead since the hill found around nine hops is smaller than for the other protocols.

## 6. Conclusions

This paper introduced F&C, a flooding algorithm for ad hoc and sensor networks that alleviates the adverse effects of traditional flooding as well as other repeal-based protocols. First, we showed that repeal-based protocols are effective only when source-target pairs are located nearby and can generate even more signaling packets compared with traditional flooding for longer routes to the resource. F&C, on the other hand, has a behavior similar to repeal-based protocols for short-route resources and similar to traditional flooding for long-route resources. F&C adopts a repeal-flooding boundary strategy that signals whether nodes should chase the initial flooding or not. This paper presented a derivation of the optimized repeal-flooding boundary and a method to estimate it in practice accurately. Various experiments were conducted in ns-3 and Simpy simulators to test F&C performance under diverse network conditions and against other repeal-based flooding protocols. The results showed that nodes in F&C can accurately estimate their optimized repeal-flooding boundary with minimal network knowledge. Overall, the results show F&C outperformed traditional flooding and other repeal-based protocols in signaling overhead and delay metrics. Finally, this paper showed that even with diverse network conditions, F&C remains a robust and efficient alternative to resource discovery protocols in wireless ad hoc and sensor networks.

## Figures and Tables

**Table 1:** Flooding contention protocols.

Algorithm	Technique	Contention Area	Flooding Speed	Chasing Technique
L-B [9]	BRIS	Unbounded	$\delta \times 2^{d-1}$	Different speeds
ERS [10]	No repletion	Bounded	δ	No chasing
LHBA [11]	BRID	Bounded	$\delta \times 2^{d-1}$	Different speeds
BERS [12]	BRIS	Bounded	$\delta \times 2^{d-1}$	Stop and wait
BERS+ [13]	BRIS	Unbounded	$\delta \times 2^{d-1}$	Stop and wait
TLRDA-C [14]	BRIS	Unbounded	mδ and nδ, m<n	Different speeds in/out the ring
BERS* [15]	BRIS	Bounded	$\delta \times 2^{d-2}$ for d>1	Stop and wait
AODV-PC [16]	BRID	Unbounded	mδ and nδ, m<n	Different speeds in/out the ring
tBERS [17]	BRID	Bounded	$\delta \times 2^{d-1}$	Stop and wait
BCIR [18]	BRID	Bounded	$\delta \times 2^{d-1}$	Stop and wait
tBERS* [17]	BRID	Bounded	$\delta \times 2^{d-2}$ for d>1	Stop and wait
BCIR* [18]	BRID	Bounded	$\delta \times 2^{d-2}$ for d>1	Stop and wait
I-BERS [19]	BRIS	Bounded	$\delta \times 2^{d-1}$	Stop and wait
ABC [20]	BRID	Unbounded	δ+md	Added delay
CMBERS+ [21]	BRID	Unbounded	$\delta \times 2^{d-2}$ for d>1	Stop and wait

**Table 2:** Repeal-flooding boundary estimation from node D seeking a route to node C.

Node	#RQ	#RP	*R*	*r*	${\mathrm{FB}}_{\mathrm{est}}$
A	6	8	7	1	5
B	11	8	9	1	6
C	0	3	2	2	1
D	3	0	2	2	1

**Table 3:** Repeal-flooding boundary estimation from node A seeking a route to node B.

Node	#RQ	#RP	*R*	*r*	${\mathrm{FB}}_{\mathrm{est}}$
A	0	16	8	8	9
B	16	0	8	8	9
C	9	7	8	2	6
D	6	10	8	2	6

**Table 4:** Repeal-flooding boundary estimation keeping the largest value of $FB_{est}$.

Node	${\mathrm{FB}}_{\max}$	${\mathrm{FB}}_{\mathrm{optimized}}$
A	9	9
B	9	9
C	6	6
D	6	6

**Table 5:** Simulation parameters.

Parameter	Value
Nodes	500
Sim area	3100 × 3100 m${}^{2}$
Tx range	250 m
Positioning	Random
Scenarios	20

**Table 6:** F&C signaling under ideal and non-ideal conditions.

	Ideal Condition	Non-Ideal Condition	
shape	65% (disk)	72% (irregular)	
density	65% (uniform)	61% (higher density)	70% (lower density)
mobility	65% (static)	60% (1 min)	75% (10 min)

## Abbreviations

The following abbreviations are used in this manuscript:

F&C	Flood and Contain
IoT	Internet of Things
ID	Identificator
BRIS	Broadcast-Repealing-Initiated in Source
BRID	Broadcast-Repealing-Initiated in Destination
TTL	Time to Live
RQ	Resource Request
RP	Resource Reply
MPR	Multipoint Relay
OLSR	Optimized Link State Routing
